# The Influence of Tobacco Use on Pulmonary Function in Elite Athletes

**DOI:** 10.3390/ijerph16193515

**Published:** 2019-09-20

**Authors:** Slavica Đorđević Šaranović, Jelisaveta Vićić, Ika Pešić, Milena Tomović, Đorđe Batinić, Milena Antić, Marijana Tadic, Sanja Mazić

**Affiliations:** 1 Serbian Institute of Sport and Sports Medicine, 11000 Belgrade, Serbia; drslavica@gmail.com (S.Đ.Š.); dj.batinic@zavod.rs (Đ.B.); m.antic@zavod.rs (M.A.); 2 Center for Sports Medicine and Exercise Therapy, Faculty of Medicine, University of Belgrade, 11000 Belgrade, Serbia; j.vicic@zavod.rs (J.V.); m.tomovic@zavod.rs (M.T.); sagabgyu@yahoo.com (S.M.); 3 Clinic for Pulmonary Diseases—Clinical Center of Serbia, 11000 Belgrade, Serbia; ika.pesic@zavod.rs; 4 Department of Internal Medicine and Cardiology, Augustenburger Platz 1, Campus Virchow-Klinikum, Charité—Universitätsmedizin Berlin, 13353 Berlin, Germany

**Keywords:** athletes, smoking, lung function, body composition

## Abstract

**Objective:** We sought to investigate the prevalence of smoking and lung function in the large cohort of elite athletes. **Methods:** This cross-sectional study included 804 athletes competing at international level who were consecutively examined from January to December 2017. Elite athletes were classified in four groups of sport disciplines (skill, power, endurance and mixed): skill (n = 141), power (n = 107), endurance (n = 105) and mixed sport disciplines (n = 451). All participants underwent pre-participation screening, including spirometry. **Results:** Study included 745 (92.7%) non-smokers, 20 (2.5%) former smokers and 39 (4.8%) active smokers. The percentage of body fat was higher and the percentage of muscle was lower in active smokers than in non-smokers and former smokers. Active smokers were more prevalent among skill and mixed than in power and endurance sports. FEV1 and FVC, as well as FEV1/FVC ratio, were significantly lower in active smokers than in non-smokers. There was no significant difference in PEF assessed in absolute values and in percentages. Forced expiratory flows, evaluated at the usual intervals (25%, 50% and 75% of FVC), were significantly lower in active smokers than in non-smokers. FEV1 and MEF25 were the lowest among active smokers in the skill sport group, whereas FEV1/FVC, MEF50 and MEF25 were the lowest among active smokers in the power sport group. In mixed and endurance disciplines there was no difference in pulmonary function between non-smokers, former smokers and active smokers. **Conclusions:** Pulmonary function was reduced in active smokers and these differences were the most prominent in skill and power sports. The percentage of body fat was the highest and percentage of muscle was the lowest in active smokers.

## 1. Introduction

The large body of evidence confirms the negative influence of tobacco smoking on cardiovascular diseases, pulmonary diseases, different types of cancers, etc. [1]. However, the limited number of studies regarding smoking habits was conducted in the population of elite athletes.

The research on the prevalence of tobacco smoking in elite athletes is very important because it represents a preventable toxic habit, which is often associated with the use of alcohol. The association between cigarette smoking and long-term reduction in physical performance [2,3], which is particularly important in elite athletes, has been previously reported.

Martinsen et al. reported that 0.8% of 677 first-year students at 16 Norwegian elite sport high schools were active smokers [4]. Veliz et al. showed that e-cigarette use (18.0%) was more prevalent during than traditional cigarette smoking (12.1%) among adolescents who participated in at least one competitive sport [5]. Yusko et al. reported very high prevalence of lifetime usage of cigarettes among student athletes—39.9% among male athletes and 35.4% among female athletes [6]. The use of smokeless tobacco during the senior year was less prevalent—32.2% among male athletes and 3.8% among female athletes [6].

Mündel et al. reported that chewing low-dose nicotine gum shortly prior to exercise significantly improved leg extensor torque but did not affect counter-movement jump height compared to a placebo [7]. The same group of authors reported that nicotine administration, whether via gum or transdermal patch, did not alter any of the psycho-physiological measures in professional and elite athletes [8]. Johnston et al. found that oral-dispersible nicotine strips increase anaerobic performance by significant elevation of cardiovascular parameters, possibly through strong sympathetic stimulation [9].

Zandonai et al. showed that muscular and cerebral oxygenation increased significantly with snus administration during an endurance exercise until exhaustion, but this did not affect fatigue perception and time to exhaustion [10]. The results showed that snus could not be considered an ergogenic substance in non-tobacco users. The same group of authors reported that regular snus use provoked more satisfaction and psychological reward than intermittent usage [11]. The investigators also revealed that 12 hours of abstinence from snus-contained nicotine affected metabolic, cardiovascular and muscular tissue oxygenation [12].

The effect of nicotine on physical performance in different studies is not consistent. Some studies observed an ergogenic effect, other an ergolytic effect, whereas the majority of studies did not reporting any change [13]. Johnston et al. in the systematic review revealed that nicotine-induced changes in physiological function could be beneficial for physical performance due to increased heart rate and blood flow [14]. However, these effects concerned only leg extensor torque and endurance performance. Other benefits were not proven.

Jang et al. included limited number of elite taekwondo athletes and performed cardiopulmonary exercise test in all of them [15]. In this small group with high prevalence of active smokers (nine non-smokers and six smokers), the authors did not find difference in maximal oxygen uptake, oxygen pulse, or exercise time. The only difference was observed in the shorter time necessary to reach the ventilatory threshold [15]. However, there are no data regarding pulmonary function in large number of elite athletes with different smoking status.

The aim of this study was to assess the prevalence of tobacco smoking in the population of Serbian elite athletes in different sport disciplines, determine the pulmonary function in these athletes and compare pulmonary function between non-smokers, former smokers and active smokers.

## 2. Methodology

This cross-sectional study included 804 elite athletes who were consecutively examined at the Serbian Institute of Sport and Sports Medicine from January to December 2017. Elite athletes were classified in four groups of sport (skill, power, endurance and mixed): skill (n = 141), power (n = 107), endurance (n = 105) and mixed sport disciplines (n = 451).

All study participants performed regular health pre-participation screening including spirometry at the institute. Inclusion criteria were: professional athletes, participating in international competitions, training regularly ≥10 h per week. Exclusion criteria were: athletes with any type of acute health condition and those who did not follow the instruction for the usage of any medication or supplements.

Anthropometric measures including body composition were taken from all the subjects included in the study and spirometry was performed in all participants. Body composition and fat percentage were measured using a body composition analyser (InBody 370, Korea). Data regarding smoking habits were obtained from each study participants. Smokers were considered all participants who used tobacco cigarette at least once a day for the last three months. There were no occasional smokers in our study. Former smokers were those participants who stopped smoking and did not smoke at least three months before our examination.

The study was approved by the Ethics Committee of the Faculty of Medicine, University of Belgrade. Informed consent was obtained from all the participants. 

### 2.1. Pulmonary Function

Pulmonary function was evaluated by a commercially available system MasterScreen Pneumo (Jaeger^TM^ pneumotach, CareFusion, Germany). The lung function measurements were always performed at the beginning of sport season (in the first two week), in the morning hours, before training or other physical effort. All participants were advised not to smoke or drink at least 8 h before examination and to have only a light breakfast at least ≥2 h before testing.

Forced expiratory manoeuvres were performed in accordance with the American Thoracic Society/European Respiratory Society (ATS/ERS) guidelines [16]. Measurements were always performed three times, and the largest forced expiratory volume at the end of the first second of forced expiration (FEV1) and forced vital capacity (FVC) from reproducible manoeuvres (i.e., between-manoeuvre differences <150 mL for FEV1 and FVC) were taken for analysis. The Global Lungs Initiatives GLI 2012 equations were used for calculation of predicted values and lower limits of normal [17]. FEV1 and FVC were also calculated as the percentage of absolute values that were obtained and predicted values for age and sex.

Direct measurements included FVC, FEV1, peak expiratory flow (PEF). The forced expiratory ratio (FEV1/FVC × 100) was calculated and presented in percentage. Maximum expiratory flow rates were evaluated at the usual intervals of 75%, 50% and 25% of exhaled forced vital capacity (MEF75, MEF50, MEF25). All parameters were measured under standard environmental conditions: comfort temperature (18–22 °C), the atmospheric pressure of 760 mmHg, and a relative atmospheric humidity of 30–60%.

### 2.2. Statistical Analysis

Continuous variables were presented as mean ± standard deviation as they showed normal distribution and they were compared using analysis of covariance (ANCOVA) because they were adjusted for age and BMI of study participants. The data distribution was examined by Kolmogorov–Smirnov’s test. Tukey post hoc analysis was used for the comparison between different groups. Differences in proportions were compared by the χ² test or Fisher test, where appropriate. All reported *p* values were two-sided. A *p*-value < 0.05 was considered statistically significant.

## 3. Results

Study included 745 (92.7%) non-smokers, 20 (2.5%) former smokers and 39 (4.8%) active smokers. There was no difference in gender distribution between these three groups. Former and active smokers were significantly older than non-smokers (Table 1). Consequently, former and active smokers trained significantly longer than non-smokers. However, there was no difference in training hours per week between three observed groups. Exercise capacity evaluated in metabolic equivalents (MET) was significantly lower in former and active smokers than in non-smokers (Table 1).

Body size and composition significantly varied between active smokers, former smokers and non-smokers (Table 1). BMI was significantly higher in active smokers than in non-smokers. The percentage of body fat was higher in active smokers than in non-smokers and former smokers (Table 1). The percentage of muscle gradually decreased from non-smokers, across active smokers, to former smokers. FEV1 and FVC in percentage were significantly lower in active smokers than in non-smokers (Table 1). FEV1/FVC ratio was significantly lower in active smokers than in non-smokers. There was no significant difference in PEF assessed in absolute values and in percentages. Maximum expiratory flow rates evaluated at the usual intervals of 75%, 50% and 25% of exhaled forced vital capacity (MEF75, MEF50, MEF25) were significantly lower in active smokers than in non-smokers (Table 1).

Comparing different sport disciplines demonstrated that elite athletes in mixed disciplines had significantly higher prevalence of non-smokers than athletes in skill, power and endurance disciplines (Table 2). Mixed disciplines had significantly higher prevalence of former smokers than power and endurance athletes. Active smokers were more prevalent among skill and mixed than in power and endurance sports (Table 2).

Pulmonary function between non-smokers, former smokers and active smokers was analysed in athletes within different sport disciplines (Table 3). In mixed and endurance disciplines there was no difference in pulmonary function between non-smokers, former smokers and active smokers.

Former and active smokers were older than non-smokers in both skill and mixed sports (Table 3). Difference in body composition existed only in skill sports, but not in mixed sports. Body fat percentage was the highest and body muscle percentage was the lowest in active smokers among athletes who belonged to the group of skill sports (Table 3). FEV1 and MEF25 were the lowest among active smokers in the skill sport group, whereas FEV1/FVC, MEF50 and MEF25 were the lowest among active smokers in the power sport group (Table 3).

## 4. Discussion

There are several important findings of this study: (i) the prevalence of former and active smokers among elite athletes was low; (ii) active smokers were more prevalent among skill and mixed than in power and endurance sport disciplines; (iii) body composition significantly varied between non-smokers, former and active smokers; (iv) FEV1, FVC and FEV1/FVC ratio, as well as MEF 25%, 50% and 75% were significantly lower in active smokers than in non-smokers.

The World Anti-Doping Agency (WADA) has inserted nicotine in its monitoring program from 2012 until 2020. The reason is laying in the fact that the effect of nicotine on exercise performance in elite athletes is still controversial [18]. Marclay et al. performed analysis of 2185 urine samples from elite athletes and assessed the prevalence of nicotine consumption. Compounds of interest in urine were detected in 23.0% participants and concentration levels corresponding to an exposure to tobacco within the last three days were found in 18.3% athletes [19]. This high amount of smokers among top athletes raised many questions and one of them was whether athletes honestly fulfilled different questionnaires. Namely, the most of studies based on questionnaire showed significantly lower prevalence of active smokers among elite athletes [4,5,6]. On the other hand, one recent study in the small group of professional male athletes in Qatar showed that prevalence of tobacco use was 27.7% [20].

Our findings showed that active and former smokers were more prevalent among skill and mixed than in power and endurance sport disciplines. This is similar to the results found in previously conducted research and other similar studies [4,5,6]. However, even though tobacco use was more prevalent in skill and mixed disciplines, pulmonary function was not necessarily lower in athletes practicing those kinds of sport. Our results showed significant difference between non-smokers, former smokers and active smokers in skill and power sports, but not in mixed sports.

BMI and percentage of fat was significantly higher, whereas the percentage of muscles was significantly lower among smokers in comparison with non-smokers in our study. Jang et al. did not find significant difference in BMI and body composition between smokers and non-smokers. However, the main limitation to reach statistical significance in this study was the small sample size (n = 15) [15]. Nicotine is related with insulin resistance [16], which is associated with change of body composition, including elevation of visceral fat. Smokers have a higher percentage of visceral fat compared to total fat than non-smokers [17]. The association between nicotine and increased visceral fat is not well understood, but it may be related to the effects of nicotine, which increases the release of cortisol and changes the equilibrium of male and female sex hormones.

The evaluation of pulmonary function in athletes who use tobacco has not been investigated extensively [21,22]. Chaabane et al. investigated 113 male professional athletes from ten ball game clubs in the same sport league in Qatar and found that athletes who used tobacco had significantly lower FEV1 and FVC than the athletes who did not use tobacco [22]. The difference in pulmonary function and deteriorated FEV1 and FVC was more pronounced among cigarette smokers than shisha smokers [22]. On the other hand, Jang et al. investigated pulmonary function by performing cardiopulmonary exercise testing in 15 taekwondo athletes and found no significant difference in parameters of ventilation or oxygen uptake during exercise or during recovery [15]. There was no difference in maximal oxygen uptake and metabolic equivalents between smokers and non-smokers [15]. Interestingly, heart rate recovery was significantly higher in smokers in the first and third minute after exercise [15]. This might indicate the slower activation of parasympathetic and higher balance of sympathetic nervous system in active smokers. Furthermore, this could suggest that the oxygen consumption of the heart muscles was increased, inducing higher heart rate and potentially lowering exercise ability. Therefore, smoking could be one of the risk factors that induce the reduction of cardiopulmonary function recovery.

Our study showed significantly lower metabolic equivalent per hour during the week in active smokers than in non-smokers, which in turn showed lower physical performance in tobacco users. Reduced physical performance in smokers was demonstrated for the first time at the middle of the last century [23]. The confirmation for this comes from the more recent study, which showed that smoking cessation significantly improved physical performance in young men [24]. Interestingly, our findings showed that lower respiratory parameters were found only in skill and power sports, but not in endurance and mixed sports. Moreover, the difference was the most prominent in MEF 50 and 25%, which indicates impairment of small airways in elite athletes who regularly used tobacco.

### Limitations

The current research has several limitations that should be mentioned. First, the information regarding tobacco use was obtained from athletes and it was not controlled quantitatively from the level of nicotine in urine or carbon monoxide level using a smokerlyzer. Therefore, the prevalence could be underestimated. Second, the influence of e-cigarettes was not evaluated because this type of smoking is not prevalent in Serbia. Third, the prevalence of active and former smokers is relatively low and in some sport disciplines very low (<5 or even 0), which made statistical analysis difficult. Fourth, our study population consisted of Caucasian elite athletes because other races are not common in our general population and, therefore, our results might be limited to white population.

## 5. Conclusions

The percentage of active smokers among elite athletes in our country is low. Body size and composition was different in athletes who are smokers from those who are non-smokers in term of higher percentage of body fat. Tobacco use did not have the same prevalence in different sport disciplines. Active smokers were more prevalent among skill and mixed than in power and endurance sports. Pulmonary function was deteriorated in smokers in comparison with non-smokers but only in skill and mixed sports, whereas in skill and endurance disciplines the difference in pulmonary function between non-smokers, former smokers and active smokers was not detected. Further follow-up studies with larger number of participants in each sport and quantitative assessment of tobacco use through urine analysis, as well as its effect on physical performance of elite athletes are necessary.

## Figures and Tables

**Table 1:** Demographic characteristics and clinical parameters in the whole study population*.

	Non-Smokers (n = 745)	Former Smokers (n = 20)	Active Smokers (n = 39)	*p*
Age (years)	22 ± 5	29 ± 6 ^d^	29 ± 8 ^a^	<0.001
Male (%)	485 (65)	15 (75)	30 (77)	0.217
Training (years)	11 ± 5	13 ± 6	15 ± 8 ^a^	<0.001
Training per week (h)	18 ± 6	20 ± 9	17 ± 7	0.210
METs	8.2 ± 2.6	6 ± 3 ^d^	6.8 ± 3.4 ^a^	<0.001
BMI (kg/m^2^)	23.1 ± 3.1	24.4 ± 2.9	25.0 ± 4.1 ^a^	<0.001
Body fat (%)	13.2 ± 5	11.7 ± 5.6	15.8 ± 8.8 ^b,c^	0.049
Body muscle (%)	48.7 ± 6.9	39.0 ± 18.1 ^d^	44.8 ± 11.8 ^a,e^	<0.001
FVC (L)	5.7 ± 1.3	5.9 ± 1.5	5.6 ± 1.4	0.710
FVC (%)	112 ± 14	112 ± 15	106 ± 13 ^b^	0.044
FEV1 (L)	4.8 ± 1.0	4.8 ± 1.1	4.5 ± 1.1	0.314
FEV1 (%)	112 ± 15	109 ± 13	102 ± 14 ^a^	<0.001
FEV1/FVC	84 ± 8	82 ± 6	80 ± 6 ^a^	0.012
PEF (L/min)	9.6 ± 2.4	10.1 ± 3.1	9,6 ± 2.1	0.615
PEF (%)	104 ± 19	103 ± 22	100 ± 17	0.323
MEF 75 (L/min)	8.0 ± 2.0	8.3 ± 2.8	7.8 ± 2	0.684
MEF 75 (%)	103 ± 21	99 ± 22	94 ± 20 ^b^	0.029
MEF 50 (L/min)	5.4 ± 1.5	5.4 ± 1.4	4.7 ± 1.5 ^b^	0.041
MEF 50 (%)	101 ± 26	97 ± 21	85 ± 25 ^a^	0.002
MEF 25 (L/min)	2.7 ± 1.2	2.4 ± 0.5	2.0 ± 0.9	0.363
MEF 25 (%)	99 ± 36	91 ± 14	76 ± 32 ^a^	0.002

BMI—body mass index; FEV1—forced expiratory volume at the end of the first second of forced expiration; FVC—forced vital capacity; MEF75, MEF50, MEF25—maximum expiratory flow rates were evaluated at the usual intervals of 75%, 50% and 25% of exhaled forced vital capacity; MET—metabolic equivalent; PEF—peak expiratory flow; ^a^—*p* < 0.01 for non-smokers vs. active smokers; ^b^—*p* < 0.05 for non-smokers vs. active smokers; ^c^—*p* < 0.05 former smoker vs. active smokers; ^d^—*p*< 0.01 for non-smokers vs. former smokers; ^e^—*p* < 0.01 for former smokers vs. active smokers; *—lung function parameters were adjusted for age and BMI.

**Table 2:** Smoking status in different sports.

	Skill (n = 125)	Power (n = 123)	Endurance (n = 105)	Mixed (n = 451)	*p*
Non-smokers (%)	103 (14)	120 (16)	105 (14)	417 (56) *	<0.001
Former smokers (%)	7 (35)	1 (5)	0 (0)	12 (60) ^#^	<0.001
Active smokers (%)	15 (38) ^	2 (5)	0 (0)	22 (57) *	<0.001

*—*p* < 0.05 for the comparison with all other sports; #—*p* < 0.01 for the comparison between mixed and power sport; ˆ—*p* < 0.05 for the comparison between skill and power sports.

**Table 3:** Demographic characteristics and clinical parameters in the athletes in skill and power sports *.

	Skill Sports				Mixed Sports			
	Non-Smokers (n = 103)	Former Smokers (n = 7)	Active Smokers (n = 15)	*p*	Non-Smokers (n = 417)	Former Smokers (n = 12)	Active Smokers (n = 22)	*p*
**Age (years)**	25 ± 7	31 ± 10 ^d^	33 ± 10 ^a^	0.001	20 ± 5	28 ± 4 ^d^	26 ± 6 ^a^	<0.001
**Male (%)**	67 (65)	6 (86)	13 (87)	0.147	266 (64)	8 (67)	16 (73)	0.685
**Training (years)**	13 ± 6	9 ± 4	16 ± 8 ^a^	0.053	11 ± 5	16 ± 5 ^d^	15 ± 7 ^a^	<0.001
**Training per week (h)**	15 ± 7	20 ± 10	17 ± 9	0.207	18 ± 6	20 ± 5	17 ± 6	0.641
**METs**	4.4 ± 2.5	3.1 ± 0.8	3.7 ± 1.8	0.186	8.3 ± 1.6	7.8 ± 2.5	8.8 ± 2.4	0.226
**BMI (kg/m^2^)**	23.2 ± 3.4	23.8 ± 1.4	25.7 ± 4.5 ^a^	0.035	23.0 ± 2.5	24.8 ± 3.5 ^f^	24.6 ± 3.9 ^a^	0.001
**Body fat (%)**	17.5 ± 7.1	9.8 ± 5.0 ^d^	20.6 ± 9.8 ^c^	0.042	12.9 ± 6.4	13.7 ± 5.6	13.0 ± 6	0.895
**Body muscle (%)**	42.0 ± 6.9	26.7 ± 12.3 ^d^	40.6 ± 12.5 ^c^	0.021	49.7 ± 4.3	49.4 ± 3.8	49.8 ± 4.0	0.972
**FVC (L)**	5.0 ± 1.0	4.8 ± 0.5	4.8 ± 1.0	0.682	5.9 ± 1.3	6.6 ± 1.6	6.3 ± 1.3	0.139
**FVC (%)**	105 ± 12	105 ± 18	99 ± 14	0.277	113 ± 15	118 ± 12	112 ± 11	0.478
**FEV1 (L)**	4.2 ± 0.8	3.9 ± 0.3	3.9 ± 0.9	0.247	5.0 ± 1.0	5.3 ± 1.2	5.0 ± 1.0	0.515
**FEV1 (%)**	104 ± 12	102 ± 12	95 ± 15 ^a^	0.032	114 ± 16	113 ± 12	107 ± 11	0.178
**FEV1/FVC**	84 ± 8	83 ± 9	80 ± 6	0.137	85 ± 8	81 ± 4	81 ± 6 ^b^	0.018
**PEF (L/min)**	9.2 ± 2.4	8.6 ± 2	9.2 ± 2.3	0.768	9.8 ± 2.5	10.8 ± 3.5	9.9 ± 2.4	0.341
**PEF (%)**	102 ± 18	93 ± 16	99 ± 17	0.474	105 ± 20	108 ± 24	100 ± 17	0.400
**MEF 75 (L/min)**	7.7 ± 2.0	7.0 ± 1.7	7.4 ± 2	0.568	8.2 ± 2.1	8.9 ± 3.2	8.2 ± 2.0	0.569
**MEF 75 (%)**	100 ± 21	89 ± 16	91 ± 21	0.199	105 ± 22	103 ± 25	96 ± 18	0.182
**MEF 50 (L/min)**	4.9 ± 1.4	4.8 ± 1.5	4.4 ± 1.7	0.508	5.6 ± 1.5	5.5 ± 1.3	5.1 ± 1.3	0.248
**MEF 50 (%)**	93 ± 24	92 ± 27	84 ± 31	0.418	104 ± 27	96 ± 15	88 ± 20 ^a^	0.013
**MEF 25 (L/min)**	2.2 ± 0.9	2.0 ± 0.5	1.6 ± 0.7 ^a^	0.015	3.0 ± 1.5	2.5 ± 0.4	2.4 ± 1.2	0.721
**MEF 25 (%)**	89 ± 31	87 ± 21	86 ± 32 ^a^	0.022	105 ± 38	91 ± 9	84 ± 33 ^b^	0.021

BMI—body mass index; FEV1—forced expiratory volume at the end of the first second of forced expiration; FVC—forced vital capacity; MEF75, MEF50, MEF25—maximum expiratory flow rates were evaluated at the usual intervals of 75%, 50% and 25% of exhaled forced vital capacity; MET—metabolic equivalent; PEF—peak expiratory flow; ^a^—*p* < 0.01 for non-smokers vs. active smokers; ^b^—*p* < 0.05 for non-smokers vs. active smokers; ^c^—*p* < 0.05 former smoker vs. active smokers; ^d^—*p* < 0.01 for non-smokers vs. former smokers; ^f^—*p* < 0.05 for non-smokers vs. former smokers; *—Lung function parameters were adjusted for age and BMI.
